# Carbamate derivatives and sesquiterpenoids from the South China Sea gorgonian *Melitodes squamata*

**DOI:** 10.3762/bjoc.8.18

**Published:** 2012-01-31

**Authors:** Li-Si Huang, Fei He, Hui Huang, Xiao-Yong Zhang, Shu-Hua Qi

**Affiliations:** 1 Key Laboratory of Marine Bio-resources Sustainable Utilization/Guangdong Key Laboratory of Marine Materia Medica/RNAM Center for Marine Microbiology, South China Sea Institute of Oceanology, The Chinese Academy of Sciences, 164 West Xingang Road, Guangzhou 510301 Guangdong, China; 2 Hainan Key Laboratory of Tropical Marine Biology and Technology, South China Sea Institute of Oceanology, The Chinese Academy of Sciences, 164 Xingangxi Road, Guangzhou 510301, China

**Keywords:** carbamate, gorgonian, *Melitodes squamata*, sesquiterpenoid

## Abstract

Five carbamate derivatives, obtucarbamates C and D (**1**, **2**), dimethyl ((carbonylbis(azanediyl))bis(2-methyl-5,1-phenylene))dicarbamate (**3**), obtucarbamates A and B (**4**, **5**), and four aromadendrane-type sesquiterpenoids, (+)-4β-*N*-methenetauryl-10β-methoxy-1β,5α,6β,7β-aromadendrane (**6**), (−)-4β-*N*-methenetauryl-10β-methoxy-1β,5β,6α,7α-aromadendrane (**7**), (−)-4α,10β-aromadendranediol (**8**), (+)-4β,10β-aromadendranediol (**9**) were obtained from the South China Sea gorgonian coral *Melitodes squamata* Nutting. Compounds **1**, **2**, **6**, and **7** were new, and their structures were established by spectroscopic analyses. Compounds **6** and **7** contained a taurine group that was rarely found in marine natural compounds, and **7** showed moderate antibacterial activity. The possible biosynthesis routes of **1**–**5** were conjectured.

## Introduction

Gorgonians are recognized to mainly produce acetogenins, sesquiterpenoids, diterpenoids, prostanoids, and steroids [1–2]. However, nitrogen-containing compounds were relatively few obtained from gorgonians. Gorgonian *Melitodes squamata* Nutting belonging to *Melithaea* family is a kind of pharmaceutical coral that has the efficacy of relieving cough, bleeding, and diarrhea, soothing nerves, and calming scare, etc [3]. A previous study on the chemical constituents of *Melithaea* family gorgonians led to the isolation of four new steroids melithasterols A–D from *Melithaea ocracea* [4].

## Results and Discussion

**Figure 1:**
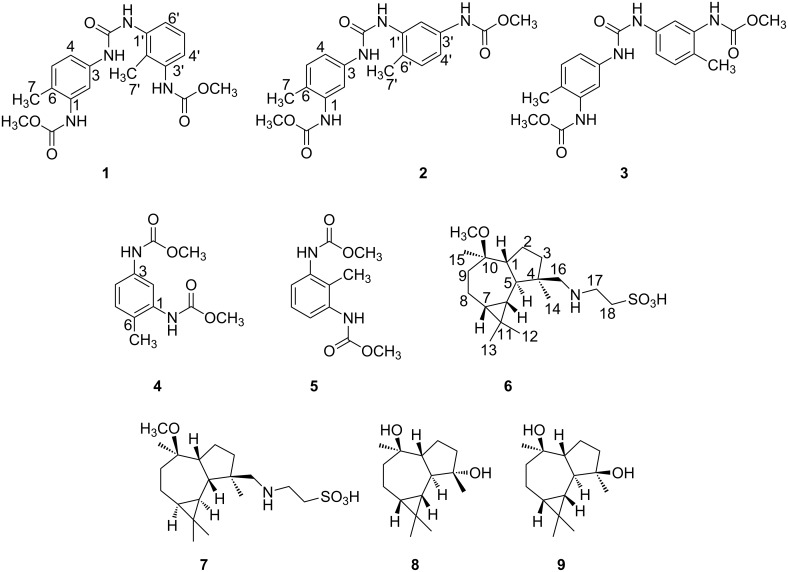
Structures of compounds **1**–**9**.

In order to further obtain new bioactive compounds from gorgonians, we studied the chemical constituents of the South China Sea gorgonian *M. squamata*, which led to the obtainment of five carbamate derivatives, obtucarbamates C and D (**1**, **2**), ((carbonylbis(azanediyl))bis(2-methyl-5,1-phenylene))dicarbamate (**3**) [5], obtucarbamate A (**4**) [6], obtucarbamate B (**5**) [6], and four aromadendrane-type sesquiterpenoids, (+)-4β-*N*-methenetauryl-10β-methoxy-1β,5α,6β,7β-aromadendrane (**6**), (−)-4β-*N*-methenetauryl-10β-methoxy-1β,5β,6α,7α-aromadendrane (**7**), (−)-4α,10β-aromadendranediol) (**8**) [7], (+)-4β,10β-aromadendranediol) (**9**) [7] (Figure 1) from the EtOH/CH_2_Cl_2_ extracts of *M. squamata*. Compounds **1**, **2**, **6**, and **7** have not been previously reported. The cytotoxicity of **1**–**9** against human malignant melanoma A735 and cervical carcinoma HeLa cell lines, and the antibacterial activity of **1**–**5** and **7** towards bacteria *Escherichia coli*, *Bacillus subtilis* and *Micrococcus luteus* were evaluated. The possible biosynthesis routes of **1**–**5** were conjectured.

Compound **1** showed the same molecular formula of C_19_H_22_N_4_O_5_ as **3**, which was determined by its HRMS–ESI (*m*/*z* 409.1568 [M + Na]^+^) and NMR spectra. The ^1^H NMR spectrum of **1** (Table 1) exhibited signals for three ABX system aromatic protons at δ_H_ 7.50 (s, 1H), 7.19 (dd, *J* = 2.0, 8.0 Hz, 1H), 7.06 (d, *J* = 8.0 Hz, 1H), three ABC system aromatic protons at δ_H_ 7.57 (d, *J* = 8.0 Hz, 1H), 7.08 (t, *J* = 8.0 Hz, 1H), 6.99 (d, *J* = 8.0 Hz, 1H), two methyl groups at δ_H_ 2.12 (s, 3H), 2.07 (s, 3H), and two methoxy groups at δ_H_ 3.63 (s, 3H), 3.64 (s, 3H). It also showed four –NH protons at δ_H_ 8.90 (s), 8.78 (s) and 8.52 (s, 2H). The ^13^C NMR and DEPT 135 spectral data (Table 1) showed the presences of 19 carbons, including three carbonyl carbons (δ_C_ 154.5, 154.6, 152.7), 12 aromatic carbons, two methyl carbons (δ_C_ 16.9, 12.4), and two methoxy carbons at δ_C_ 51.5. The NMR data of **1** were very similar to those of compounds **3**–**5** [5–6]. Compound **3** is a symmetric urea derivative that was formed from the amidation of two molecules of **4** [6]. Comparison of the NMR data of **1** with **3**–**5** suggested that **1** was an asymmetric urea derivative that was formed from the amidation of **4** and **5** [5–6].

**Table 1:** ^1^H (500 MHz) and ^13^C NMR (125 MHz) data of **1** and **2** (in DMSO-*d*_6_, δ in ppm, *J* in Hz).

C	**1**		**2**	
	δ_H_	δ_C_	δ_H_	δ_C_

1	—	136.5, C	—	136.3, C
2	7.50 (s)	114.0, CH	7.54 (s)	113.8, CH
3	—	138.3, C	—	138.0, C
4	7.19 (dd, 2.0, 8.0)	114.5, CH	7.19 (br d, 8.0)	114.0, CH
5	7.06 (d, 8.0)	130.1, CH	7.06 (d, 8.0)	130.1, CH
6	—	124.3, C	—	124.0, C
1’	—	137.1, C	—	137.5, C
2’	—	124.0, C	7.93 (s)	111.4, CH
3’	—	137.1, C	—	137.0, C
4’	7.57 (d, 8.0)	119.2, CH	7.04 (d, 8.0)	112.5, CH
5’	7.08 (t, 8.0)	125.2, CH	7.07 (d, 8.0)	129.8, CH
6’	6.99 (d, 8.0)	121.8, CH	—	121.7, C
7–CH_3_	2.12 (s)	16.9, CH_3_	2.12 (s)	17.2, CH_3_
7’–CH_3_	2.07 (s)	12.4, CH_3_	2.16 (s)	16.9, CH_3_
OCH_3_	3.64 (s)	51.5, CH_3_	3.64 (s)	51.5, CH_3_
	3.63 (s)	51.5, CH_3_	3.63 (s)	51.3, CH_3_
–NHCONH–	8.78 (s) 8.90 (s)	152.7, C	8.78 (s) 9.46 (s)	152.5, C
–NHCOO–	8.52 (s) 8.52 (s)	154.5, C 154.6, C	8.53 (s) 8.53 (s)	154.6, C 154.2, C

**Figure 2:**
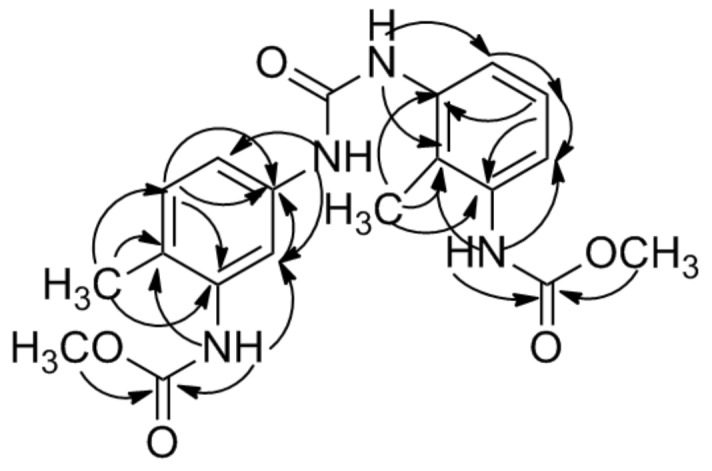
Key HMBC correlations of **1**.

The suggestion was supported by the HMBC correlations (Figure 2). In the HMBC spectrum of **1**, correlations of Me-7 (s, 2.12) with δ_C_ 130.1 (d, C-5), 124.3 (s, C-6), 136.5 (s, C-1) and Me-7’ (s, 2.07) with δ_C_ 124.0 (s, C-2’), 137.1 (s, C-1’), 137.1 (s, C-3’) indicate a methyl group attached at both C-6 and C-2’. The HMBC correlations of δ_H_ 3.64 (s) with δ_C_ 154.5 (s) and δ_H_ 3.63 (s) with δ_C_ 154.6 (s) revealed the presence of two –NHCOOMe groups. The HMBC correlations of δ_H_ 8.78 (NH) with δ_C_ 124.3 (C-6), 114.0 (C-2), δ_H_ 8.90 (NH) with δ_C_ 124.0 (C-2’), 119.2 (C-4’)_,_ and δ_H_ 8.52 (2NH) with δ_C_ 114.0 (C-2), 114.5 (C-4), 121.8 (C-6’), 124.0 (C-2’), suggest that the four –NH groups are attached at C-1, C-3, C-1’, C-3’ of two aromatic rings. Based on the above data analysis, the structure of **1** was elucidated to be as shown above and named as obtucarbamate C.

Compound **2** also exhibited the molecular formula of C_19_H_22_N_4_O_5_ as deduced from NMR and HRMS–ESI (*m*/*z* 409.1576 [M + Na]^+^). The ^1^H NMR data of **2** (Table 1) exhibited signals for two ABX aromatic systems, including six aromatic protons at δ_H_ 7.54 (s, 1H), 7.19 (br d, *J* = 8.0 Hz, 1H), 7.06 (d, *J* = 8.0 Hz, 1H), 7.93 (s, 1H), 7.07 (d, *J* = 8.0 Hz, 1H), 7.04 (d, *J* = 8.0 Hz, 1H). The ^1^H and ^13^C NMR spectral data of **2** show similarities to those of **3** [5] (Table 1). Comparison of the NMR data of **2** and **3** suggests that **2** is an asymmetric urea derivative that was formed from the amidation of two molecules of **4** [5–6], and the only difference between **2** and **3** is the amidation position of the two molecules of **4**. Based on the ^1^H NMR, ^13^C NMR HSQC, HMBC and ^1^H–^1^H COSY spectral data analysis, the structure of **2** was elucidated to be as shown above and named as obtucarbamate D.

**Figure 3:**
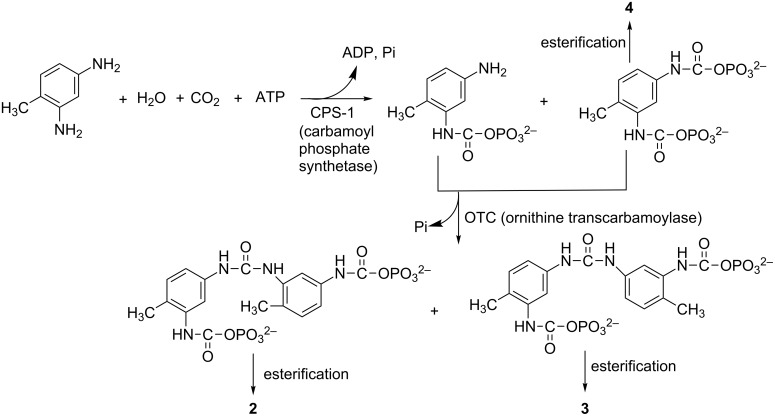
Possible biosynthesis routes of compounds **2**–**4**.

Urea derivatives are closely related in structure to carbamates. Urea is synthesized in the body of many organisms as part of the urea cycle, which is namely a cycle of biochemical reactions occurring in many animals that produces urea ((NH_2_)_2_CO) from ammonia (NH_3_). According to the reactions of the urea cycle [5], we made conjectures about the possible biosynthesis routes of compounds **2**–**4** as shown (Figure 3). The possible biosynthesis routes to **1**, **5** and **2–4** are essentially the same, except starting from 4-methylbenzene-1,3-diamine in place of 2-methybenzene-1,3-diamine. This is the first time that carbamate derivatives from gorgonians have been reported.

Compound **6** was assigned the molecular formula of C_19_H_35_NSO_4_ as deduced from NMR spectra and HRMS–ESI (*m*/*z* 372.2170 [M − H]^−^). The ^1^H NMR data (Table 2) indicate an aromadendrane skeleton with two cyclopropyl protons resonating at δ_H_ 0.44 (t, *J* = 10.0 Hz, 1H) and 0.67 (ddd, *J* = 6.5, 10.0, 11.5 Hz, 1H) [7–11]. The ^1^H NMR spectrum also showed four singlet methyl groups (δ_H_ 0.93, 1.02, 1.11, 1.11) and a methoxy group (δ_H_ 3.16). The ^13^C NMR spectrum (Table 2) displayed 19 carbon signals, including five methyl carbons (δ_C_ 16.6, 28.7, 17.6, 17.8, 48.3), seven methylene carbons (δ_C_ 24.0, 37.0, 19.5, 37.2, 45.8, 45.9, 58.9), four methine carbons (δ_C_ 50.1, 46.0, 25.6, 26.6), and three quaternary carbons (δ_C_ 19.6, 44.0, 79.1). These NMR data (Table 2) showed similarities to those of (–)-4α,10β-aromadendranediol (**8**), (+)-4β,10β-aromadendranediol (**9**) and other analogues [7–11], which suggests that **6** is an aromadendrane-type sesquiterpenoid with a methoxy group and a side chain that contained three methylene carbons.

**Table 2:** ^1^H (500 MHz) and ^13^C NMR (125 MHz) data of **6** and **7** (in CDCl_3_, δ in ppm, *J* in Hz).

No.	**6**		**7**	
	δ_H_	δ_C_	δ_H_	δ_C_

1	2.23 (dd, 8.0, 17.0)	50.1, CH	2.09 (m)	52.2, CH
2	1.73 (m)	24.0, CH_2_	1.67 (m)	25.2, CH_2_
3	1.37 (t, 11.0), 1.80 (overlap)	37.0, CH_2_	1.61 (overlap)	33.4, CH_2_
4	—	44.0, C	—	44.3, C
5	0.98 (br t, 10.0)	46.0, CH	1.72 (overlap)	41.7, CH
6	0.44 (t, 10.0)	25.6, CH	0.22 (t, 9.5)	24.3, CH
7	0.67 (ddd, 6.5, 10.0, 11.5)	26.6, CH	0.62 (m)	28.7, CH
8	0.84 (ddd, 6.5, 12.5, 13.5), 1.87 (ddd, 6.5, 11.5,13.5)	19.5, CH_2_	1.14 (m) 1.59 (m)	18.2, CH_2_
9	1.59 (t, 12.5) 1.67 (dd, 6.5, 14.5)	37.2, CH_2_	1.48 (t, 12.0), 1.83 (dd, 6.5, 14.5)	32.8, CH_2_
10	—	79.1, C	—	79.1, C
11	—	19.6, C	—	19.3, C
12	0.93 (s)	28.7, CH_3_	1.01 (s)	28.3, CH_3_
13	1.02 (s)	16.6, CH_3_	0.99 (s)	16.0, CH_3_
14	1.11 (s)	17.6, CH_3_	1.20 (s)	21.8, CH_3_
15	1.03 (s)	17.8, CH_3_	1.10 (s)	24.4, CH_3_
16	2.72 (d, 12.0), 2.94 (d, 12.0)	58.9, CH_2_	2.86 (d, 12.5), 3.04 (d, 12.5)	58.3, CH_2_
17	3.54 (t, 5.0)	45.8, CH_2_	3.27 (br s)	45.9, CH_2_
18	3.31 (t, 5.0)	45.9, CH_2_	3.44 (br s), 3.53 (br s)	45.7, CH_2_
OCH_3_	3.16 (s)	48.3, CH_3_	3.10 (s)	48.1, CH_3_

**Figure 4:**
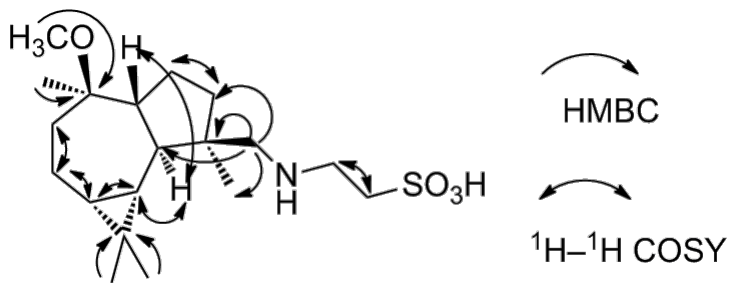
Key HMBC and ^1^H–^1^H COSY correlations of **6**.

This suggestion was proved by the HMBC correlations (Figure 4). In the HMBC spectrum of **6**, correlation of MeO– (δ_H_ 3.16, s) with δ_C_ 79.1 indicates the methoxygenation of C-10. The HMBC correlations of H-16 [δ_H_ 2.72 (d, *J* = 12 Hz), 2.94 (d, *J* = 12 Hz)] with C-3 (δ_C_ 37.0)/C-4 (δ_C_ 44.0)/C-5 (δ_C_ 46.0)/Me-14 (δ_C_ 17.6) indicate that there is a side chain at C-4 instead of a –OH group. The HMBC spectrum also shows correlations of H-16 with C-17 (δ_C_ 45.8) and H-17 [δ_H_ 3.54 (t, *J* = 5.0 Hz)] with C-16 (δ_C_ 45.8)/C-18 (δ_C_ 45.8). In the ^1^H–^1^H COSY spectrum (Figure 4), correlation of H-17 with H-18 [δ_H_ 3.31 (t, *J* = 5.0 Hz)] is observed. The above HMBC and ^1^H–^1^H COSY spectral data, combined with the molecular formula of C_19_H_35_NSO_4_, suggest the presence of a –CH_2_NHCH_2_CH_2_SO_3_H group. The suggestion was supported by the ^1^H and ^13^C NMR data comparison of the –CH_2_NHCH_2_CH_2_SO_3_H group in **6** with literature data [12–13]. The IR spectrum of **6** contains two strong bands at 1217 and 1041 cm^−1^, which supports the presence of a sulfonic acid group. The –NHCH_2_CH_2_SO_3_H group has rarely been found in marine natural compounds, such as *N*-methyltaurine, taurine, and spongidine D from sponges [12–13].

**Figure 5:**
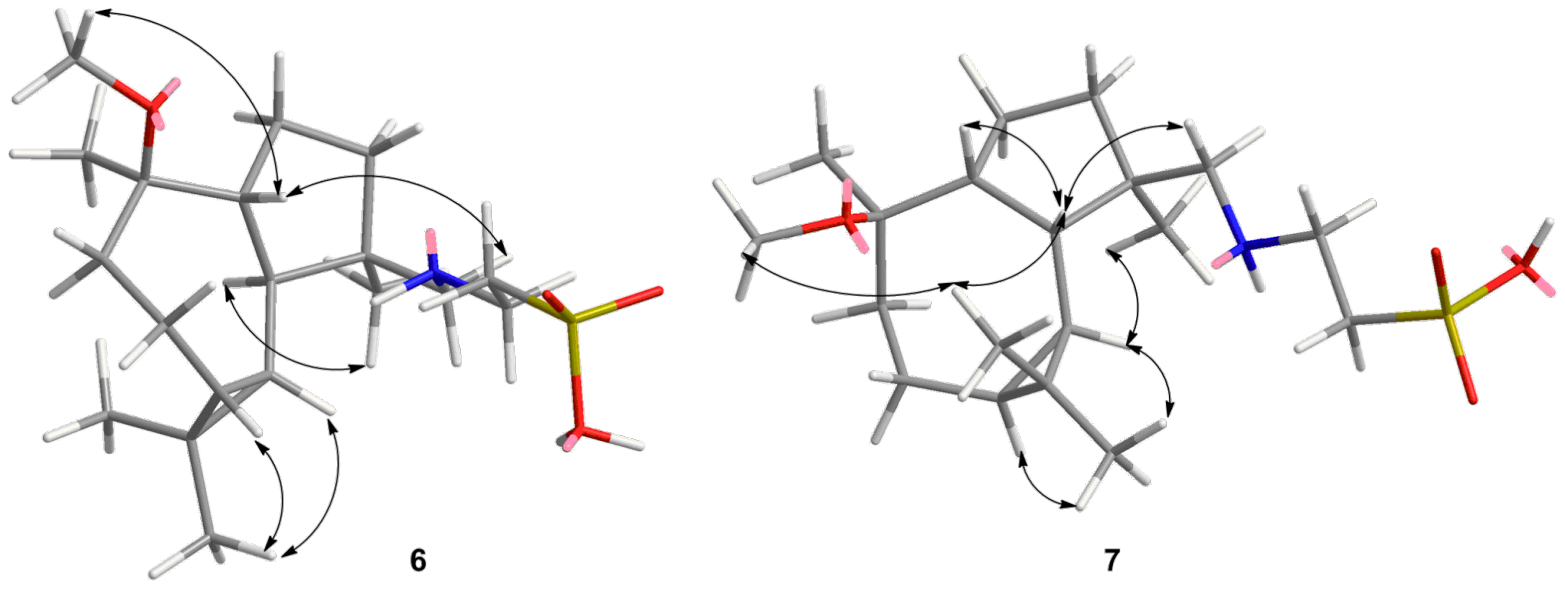
Key NOESY correlations of **6** and **7**.

The relative stereochemistry of H-1, H-5, H-6, H-7, Me-14 and Me-15 in **6** was determined by NMR data comparison with literature data and NOESY spectral analysis. The chemical shifts (δ_C_ 16.6, 28.7) of the methyl groups of Me-12 and Me-13 correlate well with those previously assigned to the corresponding methyl groups in **8**, **9** and other analogues [7–11], which suggests the *cis*-orientation of the cyclopropane ring [7–8]. The coupling constants (*J* = 10.0 Hz) between H-5 and H-1/H-6 indicate the *trans-*relationship of H-5 with H-1 and H-6. By comparison with the known chemical-shift data and shift parameters [7–11], the β-orientation of H-1, H-6, H-7 and α-orientation of H-5 were assigned. In the seven-numbered ring, the methyl group C-15 (δ_C_ 17.8) appears at a high field, reflecting that Me-15 is not in the same orientation as H-6 and H-7 [7]. In the NOESY spectrum, correlations of OMe-10 with H-1, H-6, H-7 are observed (Figure 5), which suggest that OMe-10, H-1, H-6, H-7 are in β-orientation, meanwhile, the presence of correlations between H-5 and Me-14/Me-13 indicates that H-5, Me-14, Me-13 are in α-orientation. Thus, the structure of **6** was elucidated to be as shown and named as (+)-4β-*N*-methenetauryl-10β-methoxy-1β,5α,6β,7β-aromadendrane.

Compound **7** showed the same molecular formula of C_19_H_35_NSO_4_ as **6** on the basis of NMR spectra and HRMS–ESI (*m*/*z* 372.2172 [M − H]^−^). The ^1^H and ^13^C NMR spectral data of **7** (Table 2) showed similarities to those of **6**, except for some obvious differences in the chemical shift data of H-5 (from δ_H_ 0.98 to δ_H_ 1.72), C-3 (from δ_C_ 37.0 to δ_C_ 33.4), C-5 (from δ_C_ 46.0 to δ_C_ 41.7), C-9 (from δ_C_ 37.2 to δ_C_ 32.8), and C-15 (from δ_C_ 17.8 to δ_C_ 24.4), which were caused by the stereochemistry change. In the ^1^H–^1^H COSY spectrum, no correlation between H-1 and H-5 is observed, which suggests a small coupling constant and the *cis* relationship between H-1 and H-5. The coupling constant (*J* = 9.5 Hz) between H-5 and H-6 indicates the *trans-*relationship of H-5 with H-6. In the NOESY spectrum, H-16 shows correlations with H-1/H-5, which suggests that H-16, H-1, and H-5 are in β-orientation; meanwhile, correlations of H-6 with H-7/Me-14/Me-13 indicate that H-6, H-7, Me-14 and Me-13 are in α-orientation. The chemical shift of Me-15 (δ_C_ 24.4) appears at a slightly lower field reflecting that Me-15 is at the same α-orientation as H-6 and H-7 [7]. Thus, the structure of **7** was elucidated to be as shown and named as (−)-4β-*N*-methenetauryl-10β-methoxy-1β,5β,6α,7α-aromadendrane.

The cytotoxicity of compounds **1**–**9** against human malignant melanoma A735 and cervical carcinoma HeLa cell lines was evaluated by MTT assay. The results show that **1**–**9** does not exhibit cytotoxicity against A735 and HeLa cell lines with IC_50_ > 200 μg/mL. Antibacterial activity of compounds **1**–**5** at a concentration of 25 μg/disc (diameter 6 mm) was measured against bacteria *Escherichia coli*, *Bacillus subtilis*, and *Micrococcus luteus* strains by using standard disc-diffusion assay. The results show that **1**–**5** exhibit no inhibitory effects towards all tested bacteria at 25 μg/disc, **7** can inhibit the growth of *B. subtilis* and *M. luteus* with inhibition zones of 6.3 mm and 6.2 mm, respectively, at 25 μg/disc, while **7** has no effect towards *E. coli* at 25 μg/disc. It was reported that taurine and its derivatives have a number of physiological functions, including interference with GABA and glycine receptors, antinociceptic effects, anticonvulsive actions, neuroprotective actions, etc. [14]. In this study, because of the limited quantity of compound, we did not further test the other bioactivities of **6** and **7**.

## Experimental

### General experimental procedures

Optical rotations were measured with a Horiba SEAP-300 spectropolarimeter. UV spectra were measured with a Shimadzu double-beam 210A spectrophotometer in MeOH solution. ^1^H, ^13^C NMR and 2D NMR spectra were recorded on a Bruker AV-500 MHz NMR spectrometer with TMS as internal standard. MS spectral data were obtained on an LCQDECA XP HPLC/MSn spectrometer for ESIMS. Silica gel (200–300 mesh) for column chromatography and GF_254_ for TLC were obtained from the Qindao Marine Chemical Factory, Qindao, People’s Republic of China.

### Animal material

The South China Sea gorgonian coral *Melitodes squamata* Nutting was collected in Sanya, Hainan province, China in July 2008 and identified by Prof. Huang H., the South China Sea Institute of Oceanology, *Academia Sinica*. A voucher specimen (No. 0802) was deposited in the South China Sea Institute of Oceanology, *Academia Sinica*, Guangzhou, China.

### Extraction and isolation

The frozen specimen of the South China Sea gorgonian coral *M. squamata* (20 kg, wet weight) was extracted with EtOH/CH_2_Cl_2_ (2:1) three times at room temperature and the solvent was evaporated in vacuo. The residue was partitioned in H_2_O and extracted with EtOAc and *n*-BuOH three times each. The extracts of each respective solvent were combined. The EtOAc and *n*-BuOH extracts were concentrated in vacuo to afford 35.17 g and 16.85 g of residue, respectively. The EtOAc extract was subjected to silica-gel column chromatography (column A), eluted with CHCl_3_/MeOH (from 1:10 to 0:10). By combining the fractions with TLC (GF_254_) monitoring, 17 fractions were obtained. Fraction A-5 was applied to a Sephadex LH-20 column (column B), eluted with CHCl_3_/MeOH (1:1), to obtain four fractions. Fraction B-4 was further purified by HPLC (MeOH/H_2_O 45:100) to yield **1** (1.8 mg, *t*_R_ = 23.5 min), **2** (1.8 mg, *t*_R_ = 41.2 min), **3** (2.8 mg, *t*_R_ = 38.2 min), **4** (2.7 mg, *t*_R_ = 13.8 min) and **5** (1.8 mg, *t*_R_ = 9.0 min), separately. Fraction B-3 was further purified by RP-18 reverse-phase silica-gel column chromatography (column C), eluted with MeOH/H_2_O (30:100), to yield **8** (14.8 mg) and **9** (9.5 mg). Fraction A-11 was separated by a Sephadex LH-20 column (column D), eluted with CHCl_3_/MeOH (1:1) to give five fractions. Fraction D-2 was subjected to silica-gel column chromatography (column E), eluted with CHCl_3_/MeOH (10:1), to yield **6** (4.2 mg) and three other fractions. Fraction E-2 was furthered purified by a Sephadex LH-20 column (MeOH/H_2_O 95:5) to yield **7** (4.8 mg).

**Obtucarbamate C (1):** White powder; UV (MeOH): 258, 216 nm; ^1^H and ^13^C NMR data, see Table 1; (+)-ESIMS (*m*/*z*): 409.0 [M + Na]^+^, 795.0 [2M + Na]^+^; HRMS–ESI (*m*/*z*): [M + Na]^+^ calcd for C_19_H_23_N_4_O_5_Na, 409.1590; found, 409.1568.

**Obtucarbamate D (2):** White powder; UV (MeOH): 258, 216 nm; ^1^H and ^13^C NMR data, see Table 1; (+)-ESIMS (*m*/*z*): 409.1 [M + Na]^+^, 795.3 [2M + Na]^+^; HRMS–ESI (*m*/*z*): [M + Na]^+^ calcd for C_19_H_23_N_4_O_5_Na, 409.1590; found, 409.1576.

**(+)-4β-*N*-methenetauryl-10β-methoxy-1β,5α,6β,7β-aromadendrane (6):** White powder; [α]_D_^20^ +26.4° (*c* 0.28, MeOH); ^1^H and ^13^C NMR data, see Table 2; (−)-ESIMS (*m*/*z*): 372.1 [M − H]^−^, 745.4 [2M − H]^−^; HRMS–ESI (*m*/*z*): [M − H]^−^ calcd for C_19_H_34_NSO_4_, 372.2209; found, 372.2170.

**(−)-4β-*N*-methenetauryl-10β-methoxy-1β,5β,6α,7α-aromadendrane (7):** White powder; [α]_D_^20^ −7.5° (*c* 0.48, MeOH); ^1^H and ^13^C NMR data, see Table 2; (−)-ESIMS (*m*/*z*): 372.2 [M − H]^−^, 745.4 [2M − H]^−^; HRMS–ESI (*m*/*z*): [M − H]^−^ calcd for C_19_H_34_NSO_4_, 372.2209; found, 372.2172.

## References

[1] Coll J C (1992). Chem Rev.

[2] Rodríguez A D (1995). Tetrahedron.

[3] Shao C L, Fu X M, Wang C Y, Han L, Liu X, Fang Y C, Li G Q, Zeng X Q, Liu G X, Guan H S (2009). Period Ocean Univ China.

[4] Kobayashi M, Kanda F (1991). J Chem Soc, Perkin Trans 1.

[5] Brown G W, Cohen P P (1960). Biochem J.

[6] Kuo Y H, Jou M H (1990). Chem Express.

[7] Goldsby G, Burke B A (1987). Phytochemistry.

[8] Bohlmann F, Grenz M, Jakupovic J, King R M, Robinson H (1983). Phytochemistry.

[9] Beechan C M, Djerassi C, Eggert H (1978). Tetrahedron.

[10] Jizba J, Laudová V, Samek Z, Ubik K, Novotny L (1981). Collect Czech Chem Commun.

[11] Liu H-J, Wua C-L, Becker H, Zapp J (2000). Phytochemistry.

[12] De Marino S, Iorizzi M, Zollo F, Debitus C, Menou J-L, Ospina L F, Alcaraz M J, Payá M (2000). J Nat Prod.

[13] Xynas R, Capon R J (1989). Aust J Chem.

[14] Simo S, Oja P S (2007). Proc West Pharmacol Soc.

